# Drug Reaction With Eosinophilia and Systemic Symptoms (DRESS) Syndrome Following Initiation of Sulfasalazine for Ulcerative Colitis: A Yearlong Clinical Challenge

**DOI:** 10.1155/crgm/8870613

**Published:** 2025-06-14

**Authors:** Michael B. Andrews, Michael V. Patrone, Stephen J. Bickston

**Affiliations:** ^1^Department of Internal Medicine, Virginia Commonwealth University, Richmond, Virginia, USA; ^2^Division of Gastroenterology, Hepatology and Nutrition, Virginia Commonwealth University, Richmond, Virginia, USA

## Abstract

Sulfasalazine, a commonly prescribed medication for treating ulcerative colitis, can cause a severe yet rare adverse drug reaction known as drug reaction with eosinophilia and systemic symptoms (DRESS) syndrome. In this case, a female patient experienced a severe flare of her ulcerative colitis, accompanied by sulfasalazine-induced DRESS syndrome. The ulcerative colitis flare and acute liver injury resolved with a treatment regimen of high-dose steroids and cyclosporine. However, she continued to have treatment-resistant dermatologic changes and delayed organ involvement, including the development of hyperthyroidism that required close multidisciplinary follow-up. IVIG was used to successfully wean steroids and cyclosporine after a year of therapy.

## 1. Introduction

Drug reaction with eosinophilia and systemic symptoms (DRESS) syndrome or drug-induced hypersensitivity syndrome (DIHS) is a rare and potentially life-threatening adverse drug reaction. It has a reported prevalence of 2 per 100,000 patients and a mortality rate of 10%, often secondary to fulminant hepatic failure [1, 2]. DRESS/DIHS classically presents with multiorgan involvement. DRESS/DIHS typically presents two to six weeks after initiation of the culprit medication, although it can occur earlier with re-exposure [2, 3]. The most common medications associated with DRESS/DIHS include antiepileptics (lamotrigine, phenytoin, and carbamazepine), antibiotics (vancomycin, trimethoprim-sulfamethoxazole, and minocycline), and allopurinol [3, 4]. This case report highlights a patient with ulcerative colitis (UC) on sulfasalazine who presented with DRESS/DIHS in the midst of a UC flare.

## 2. Case Report

A 34-year-old female with pancolitis UC was prescribed sulfasalazine 1000 mg daily for treatment of UC. Labs prior to initiation of sulfasalazine were unremarkable with a white blood cell (WBC) count of 8.42 (3.9–11.7 10e9/L), aspartate transaminase (AST) of 14 (0–50 U/L), alanine transaminase (ALT) of 7 (0–50 U/L), alkaline phosphatase (ALP) of 58 (40–120 U/L), and total bilirubin of 0.1 (0–1.3 mg/dL).

Five weeks later, she presented with intermittent high-grade fevers, chills, bloody diarrhea, and the development of a pruritic rash. The rash first appeared on her neck and then spread to her upper torso and face.

**Figure 1:**
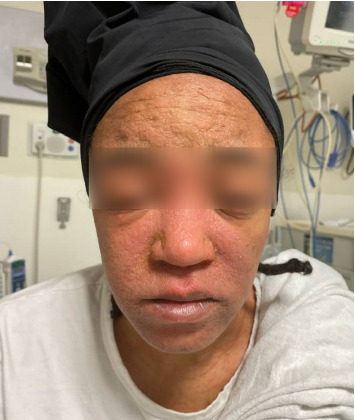
Facial edema with pruritic coalescing erythematous macules and papules on her chest.

**Figure 2:**
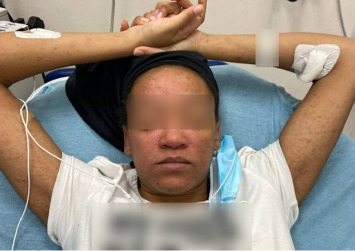
Pruritic coalescing erythematous macules and papules on her face and arms.

**Figure 3:**
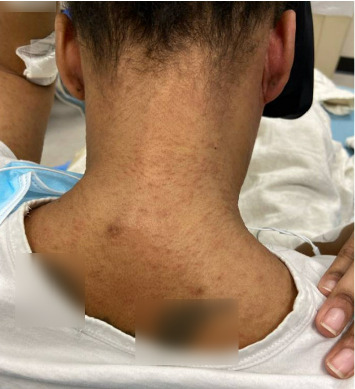
Pruritic coalescing erythematous macules and papules on her neck.

**Figure 4:**
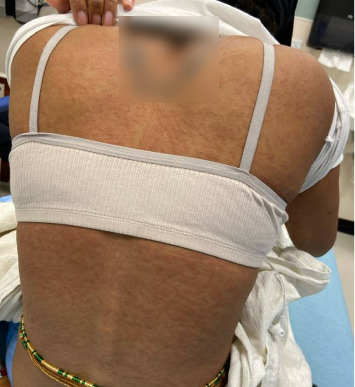
Pruritic coalescing erythematous macules and papules on her back.

On admission, she was febrile (39.7°C), tachycardic, and appeared uncomfortable but had no signs of altered mental status. Facial edema and cervical lymphadenopathy were present (Figures 1 and 2). Skin exam was notable for pruritic coalescing erythematous macules and papules on her chest, abdomen, back, arms, and thighs (Figures 2, 3, and 4).

Labs revealed a WBC count of 23.9 (3.9–11.7 10e9/L) with absolute eosinophils of 1.1 (0–0.4 10e9/L), elevated AST of 291 (0–50 U/L), ALT of 641 (0–50 U/L), ALP of 214 (40–120 U/L), total bilirubin of 2.0 (0–1.3 mg/dL), albumin of 3.4 (3.7–5.2 g/dL), INR of 1.5, creatinine (Cr) of 0.66 (0.5–1.0 mg/dL), and C reactive protein (CRP) of 9.30 (0–0.5 mg/dL). Blood cultures were negative. Antinuclear antibody (ANA) was negative with a titer of 1:80. Additional DRESS/DIHS-related infectious serologies included *Mycoplasma pneumoniae* Ab IgG 363 (positive > 320), *Mycoplasma pneumoniae* Ab IgM 1013 (positive > 950), human herpes virus 6 IgG 1.30 (positive > 1.10), human herpes virus 6 IgM < 1:10 (positive > 1.10), and undetectable human herpes virus 6, Epstein–Barr virus (EBV), and cytomegalovirus (CMV) quantitative DNA PCR.

Further acute liver injury workup ruled out viral, autoimmune, vascular (via liver Dopplers), and biliary etiologies. There was no identifiable preceding hypotensive episode to suggest ischemic etiology of the liver injury. Drug-induced liver injury was also considered as the patient was prescribed adalimumab injections for treatment of UC but had not started this medication nor was she taking any other medications, aside from sulfasalazine, within the months leading up to her presentation. CT abdomen/pelvis with contrast and MRI with magnetic resonance cholangiopancreatography (MRCP) revealed diffuse colitis without biliary abnormalities. Stool studies showed fecal leukocytes, stool calprotectin 1070 (0–120 μg/g), and stool pathogen direct testing was negative for *Clostridium difficile*, *Salmonella*, *Campylobacter*, *Shigella*, enteroaggregative, enteropathogenic, and enterotoxigenic *Escherichia. coli*.

Dermatology was consulted for co-management of DRESS/DIHS. Treatment involved escalating doses of steroids and cyclosporine until laboratory values stabilized. Maximum therapeutic doses included intravenous methylprednisolone 500 mg per day in two divided doses and oral cyclosporine 5 mg/kg/day in two divided doses. One day of single-strength N-acetylcysteine (NAC) infusion was administered when AST and ALT reached 1000 U/L.

Labs peaked in the following order: absolute eosinophils 3.0 (0–0.4 10e9/L) on hospital Day 2; CRP 9.9 (0–0.5 mg/dL) on hospital Day 3; WBC count 30.3 (3.9–11.7 10e9/L) on hospital Day 4; ALP 265 (40–120 U/L), total bilirubin 2.3 (0–1.3 mg/dL), and INR 1.8 on hospital Day 7; and AST 1348 (0–50 U/L) and ALT 1847 (0–50 U/L) on hospital Day 8. She was discharged on hospital Day 11 on a prednisone and cyclosporine taper with close subspecialty follow-ups.

Prednisone was tapered by 5 mg weekly from 60 mg daily at discharge to 50 mg at the time of her dermatology follow-up appointment. Cyclosporine was tapered by 50 mg weekly from 150 mg twice daily at discharge to 50 mg twice daily at the time of her dermatology follow-up appointment. At her follow-up appointment a few weeks after discharge, her prednisone was tapered to 40 mg, and cyclosporine was discontinued. Over the next week, she experienced a recurrence of the rash and fever which prompted re-admission. Immunosuppressive therapy was escalated to prednisone 50 mg daily and cyclosporine 150 mg twice daily. She was discharged on this regimen given there were no signs of internal organ damage.

At her seven-month follow-up appointment, she remained on cyclosporine and high-dose prednisone. Additional attempts to wean therapy were limited by rash recurrence despite her UC being successfully treated with adalimumab. However, she later developed biopsy-proven CMV colitis and was treated with valganciclovir. A few months later, she developed symptomatic hyperthyroidism and was referred to endocrinology. She experienced tremors with an elevated free thyroxine (T4) and undetectable thyroid-stimulating hormone (TSH).

Dermatology continued to follow her closely in the clinic and added a split dose treatment of intravenous immunoglobulin (IVIG) for relapsing DRESS/DIHS with symptomatic improvement at the nine-month follow-up. Finally, after a year since her initial presentation, she was successfully tapered off prednisone and cyclosporine without recurrence of symptoms.

## 3. Discussion

This case report describes a typical presentation of a rare adverse drug reaction following five weeks of sulfasalazine for the treatment of UC. Overall, major clinical improvement occurred within a week of inpatient treatment with high-dose steroids and cyclosporine. Unfortunately, despite improvement in her UC and acute liver injury, it took one year to completely taper off high-dose steroids and cyclosporine without symptom recurrence. She continues to require close multidisciplinary monitoring with many specialists.

As illustrated in this case, the classic presentation of DRESS/DIHS starts with a fever, followed by the development of a diffuse, pruritic, erythematous, morbilliform rash, facial edema, and lymphadenopathy [2, 5]. The most common laboratory findings include leukocytosis with eosinophilia and elevated liver enzymes with a hepatocellular pattern [6]. This case also highlights the importance of prompt identification of the adverse drug reaction, removal of the culprit medication, and multidisciplinary collaboration with timely initiation of treatment. Patients requiring systemic corticosteroids are at increased risk for CMV reactivation and other systemic infections [7]. Careful long-term monitoring for both infectious complications as well as delayed extracutaneous manifestations is warranted.

DRESS/DIHS may also be accompanied by less common gastrointestinal manifestations including esophagitis, gastritis, enteritis, colitis, and, in rare cases, pancreatitis. These can mimic a UC flare in patients with a prior history of UC and may be associated with the reactivation of viral pathogens such as CMV [8].

In addition, DRESS/DIHS may present similarly to and/or be on the same disease spectrum as another highly fatal systemic condition, macrophage activation syndrome (MAS) or secondary hemophagocytic lymphohistiocytosis (HLH) [9]. Both commonly present with fever, rash, and end-organ damage. However, in contrast to DRESS/DIHS, MAS or secondary HLH is more rare, has a higher mortality rate (20%–50%), and occurs due to cytokine storming from hyperactivation of T-cells and macrophages [9–11]. Triggers often include malignancy, viral infections, and rheumatologic or other autoimmune diseases [11]. It is characterized by additional laboratory abnormalities such as hyperferritinemia, hypertriglyceridemia, hypofibrinogenemia, and cytopenias [9]. Research assessing the potential overlap of these syndromes is ongoing with one hypothesis suggesting persistent or untreated DRESS may lead to secondary HLH, though more investigation is needed [9, 11]. Therefore, it is important to consider progression to MAS or secondary HLH in a clinically deteriorating patient despite removal of the culprit medication and appropriate first-line therapy.

Lastly, to prevent anchoring bias, the differential diagnosis for a patient with UC and the constellation of findings described in this case should remain broad. These include alternative severe cutaneous adverse drug reactions (Stevens–Johnson syndrome and toxic epidermal necrolysis), other viral infections (EBV and CMV), autoimmune conditions (systemic lupus erythematosus and vasculitis such as leukocytoclastic vasculitis [LCV]), and malignancy (lymphoma) [12].

## 4. Conclusion

This case highlights a yearlong clinical challenge managing a rare but severe adverse drug reaction following the use of sulfasalazine to treat UC. Early identification, prompt medication discontinuation, and a multidisciplinary treatment approach consisting of high-dose steroids, cyclosporine, and, if necessary, IVIG, are imperative.

## Data Availability

Data sharing is not applicable to this article as no new data were created or analyzed in this study.
